# 

**Published:** 1891-06-13

**Authors:** 


					ISINGLASS
AND INVALIDS.
0
t
h for INFANTS
No. 246. Vol. X.
Saturday,
June 13th, 1891.
[One Penny.

				

